# The Neural Correlates of Humor Creativity

**DOI:** 10.3389/fnhum.2016.00597

**Published:** 2016-11-25

**Authors:** Ori Amir, Irving Biederman

**Affiliations:** ^1^Department of Psychology, University of Southern California, Los AngelesCA, USA; ^2^Media Neuroscience Lab, Department of Communication, University of California, Santa Barbara, Santa BarbaraCA, USA; ^3^Neuroscience Program, University of Southern California, Los AngelesCA, USA

**Keywords:** humor creation, fMRI, creativity, expertise, comedians, cartoon captions, temporo-occipital junction (TOJ), medial prefrontal cortex (mPFC)

## Abstract

Unlike passive humor appreciation, the neural correlates of real-time humor creation have been unexplored. As a case study for creativity, humor generation uniquely affords a reliable assessment of a creative product’s quality with a clear and relatively rapid beginning and end, rendering it amenable to neuroimaging that has the potential for reflecting individual differences in expertise. Professional and amateur “improv” comedians and controls viewed New Yorker cartoon drawings while being scanned. For each drawing, they were instructed to generate either a humorous or a mundane caption. Greater comedic experience was associated with decreased activation in the striatum and medial prefrontal cortex (mPFC), but increased activation in temporal association regions (TMP). Less experienced comedians manifested greater activation of mPFC, reflecting their deliberate search through TMP association space. Professionals, by contrast, tend to reap the fruits of their spontaneous associations with reduced reliance on top-down guided search.

## Introduction

A handful of studies have recently begun exploring the neural correlates of creativity, with tasks ranging from narrative generation (Howard-Jones et al., 2005) to jazz improvisation (Limb and Braun, 2008) to creative drawing (Schlegel et al., 2015). Unfortunately, the cortical regions reported by the various studies to be associated with “creativity” were as diverse as the tasks employed, save for the often observed involvement of the prefrontal cortex (Dietrich and Kanso, 2010). It has been suggested that activation of the medial prefrontal cortex (mPFC) and a deactivation of the dorsolateral prefrontal cortex (dlPFC) were the hallmarks of creative processing, along with regions associated with the particular type of creative task (e.g., Limb and Braun, 2008; Liu et al., 2012, 2015). However, a one-dimensional comparison between creative and non-creative control conditions (e.g., jazz improvisation vs. playing from memory; Limb and Braun, 2008) may be inadequate for revealing the roles played by different brain regions in a creative endeavor, as it can only reveal a set of regions, typically unsurprising (e.g., visual regions for book cover design, Ellamil et al., 2012; language regions for poetry composition, Liu et al., 2015), associated with a particular creative task (as well as, commonly, the mPFC). Exploring two additional dimensions of a creative domain can further enhance its value as a testbed for the study of creativity: quality and expertise. With humor, the quality of the creative product (i.e., funniness) can be easily evaluated by a spontaneous laugh as well as a readily generated judgment. Although the laugh reflects a subjective state, it is one that is readily accessible for ratings and typically has high agreement across individuals. Unlike the study of passive humor appreciation (e.g., Goel and Dolan, 2001; Watson et al., 2007; Samson et al., 2008; Chan et al., 2013; Vrticka et al., 2013; Amir et al., 2015), the rarity and spontaneous origin of humor creation have rendered that domain an unlikely target of fMRI investigation (Martin, 2010). To meet this challenge, we recruited professional “improv” comedians who routinely generate humorous ideas rapidly and on cue.

Previous studies of creativity rarely examined expertise effects, since often the tasks have no experts, e.g., generate alternative uses of objects, and others would be too challenging for a control group, e.g., improvise jazz. Imaging studies comparing experts to non-experts are typically limited to perceptual/technical judgments requiring no creativity (e.g., Calvo-Merino et al., 2005; Kirk et al., 2009). Generating humorous ideas, however, is a task nearly anyone can attempt, and participants with different levels of expertise/talent can be identified. Thus far, only the acts of poetry composition and creative drawing have been studied with the aim of determining the neural correlates of both quality and expertise (Liu et al., 2015; Schlegel et al., 2015). Finally, a humorous creation based on a particular stimulus—a captionless cartoon in the present study–affords a natural and tighter control than most creativity studies: the generation of a mundane statement that would be appropriate for the same cartoon drawing. Such a control allows a distinction between standard problem solving and creative thinking (Mednick, 1962).

## Materials and Methods

**FIGURE 1:**
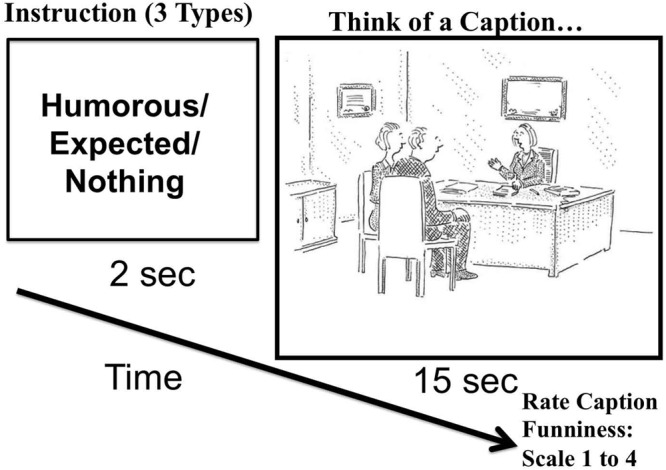
**The time-course of a trial.** Participants were prompted to think of a Humorous (HUM), Expected (MUN), or no caption. Try quickly to think of a funny caption, before you continue reading the following sample caption by one of the participants during the HUM condition: “So this is awkward. I am the woman your husband has been cheating with. Either way it will be $200 for the marriage counseling...” Original cartoon by Robert Mankoff © Published on September 23, 2002, in the NewYorker magazine, and was modified from the original for the experimental task so that all text was removed (in the original drawing the diploma on the wall read “Marriage Counselor”). Adapted and modified with permission.

**FIGURE 2:**
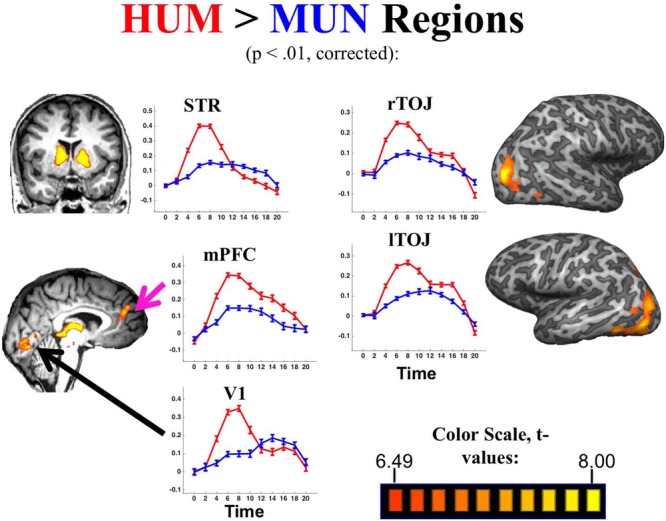
**Regions with higher activation for HUM (red) vs. MUN (blue).** Graphs depict percent BOLD signal change over time (sec).

Participants underwent fMRI scanning while looking at a series of cartoon drawings, minus the captions, of human interactions in various contexts (e.g., office, cocktail party; see **Figure 1**), that originally appeared in the NewYorker Magazine. In a post-session debriefing, in which participants described an introspection of their creative process during the experiment, none of the particpants reported familiarity with the drawings. In order to isolate active humor generation from any effects of passive humor appreciation, we selected drawings that were not funny by themselves. The captions that originally accompanied the drawings and all other text were removed, and some drawings were processed with Photoshop to remove elements that were inherently funny. Prior to the presentation of each cartoon, subjects were cued to generate (a) a humorous caption, (b) a mundane caption or (c) no caption (**Figure 2** shows the overall activation for the contrast of the conditions (a)–(b)). Each participant rated on a 4-point scale how funny their caption was on each trial. At the end of the scanning, participants were asked to recall as many of the captions as they could. Their recall was cued by presentation of the drawings. Independent ratings of those recalled captions were made by other raters (students at the University of Southern California) who judged the funniness of the recalled captions in the context of the drawings, allowing us to compare the neural correlates of successful vs. unsuccessful humor generation.

### Participants

**FIGURE 3:**
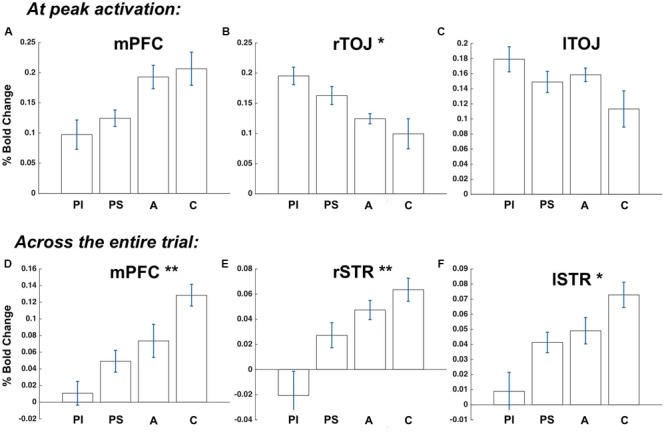
**Group differences among Professional Improv Comedians (PI), Professional Stand-Ups (PS), Amateurs (A)** and Controls **(C)**. **(A–C)** bars represent average HUM minus MUN activation during its peak; **(D–F)** bars represent activation averaged across the entire trial. Significance of expertise effect is represented by ^∗^*p* < 0.05, ^∗∗^*p* < 0.01.

The participants constituted three groups:

(a) Professional Comedians (13 individuals, mean age 35.4, range: 26–47; one female). Six were members of the renowned Los Angeles’ “Groundlings” improv troupe and seven were professional stand-up comedians, all of whom write their own material, with significant stand-up related TV credits (e.g., multiple late night show appearances, stand-up specials). (The proximity of USC to Hollywood facilitated the recruitment of these professional and promising amateur comedians.) No significant differences were observed in the pattern of activity of Professional Improv or Stand-Up Comedians, so the two groups were collapsed into a Professionals’ group, in all but one of the analyses. That exception is shown in **Figure 3**: bar graphs of group differences in selected ROIs, and the associated analysis.

(b) Nine promising Amateur Comedians (Mean age 27.2, range: 20–33; two females) each with several years of experience in stand-up and/or improv, who demonstrated a significant potential for developing into a professional comedian relative to their peers. While the amateurs’ focus may be in either stand-up or improv, all had at least some experience with both, so they are treated here as one group.
(c) Eighteen Controls (Mean age 24.9, range: 19–34; 7 females). Controls were all either honor students, graduate students or faculty at the University of Southern California, selected to roughly match the high intelligence reported for successful comedians (Greengross et al., 2012). Age and sex effects have been statistically assessed in all group comparisons.

The study was approved by the Institutional Review Board of the University of Southern California and the participants all signed informed consent.

### Procedure

Each trial (**Figure 1**) lasted 17 s. For the first 2 s of a trial, a single word cued the desired caption type: (1) “Humorous” was the cue for the participants to think of a funny caption (caption = something one of the characters would say) for the drawing (Humor [HUM] condition); (2) “Expected” was the cue to think of a caption that would fit the drawing but be mundane and expected (Mundane [MUN] condition); (3) “Nothing” (NOTH condition) was the cue look at the drawing without thinking of a caption. Then a drawing depicting a human interaction appeared at the center of the screen (subtending a visual angle of ∼8°). In the HUM and MUN conditions, participants had 15 s to generate a caption for the drawing and rate it for funniness. Once participants thought of a caption they were instructed to immediately rate it using a keyboard, on a 4-point scale (1 – not funny, 2 – a little funny, 3 – pretty funny, 4 – very funny). Each participant saw each drawing once, and drawings were approximately counterbalanced across the three conditions between participants. (The balancing was approximate as the number of participants was not divisible by the number of conditions for some groups). Each run lasted 7.9 min with 24 jittered trials sequenced such that each sequence of two conditions appeared the same number of times. Most participants completed six runs; all completed at least four. No runs or participants were discarded. Presentation sequences were programmed with Psychophysics Toolbox (Brainard, 1997; Pelli, 1997) running on MATLAB (The MathWorks, Natick, MA, USA).

### Data Acquisition

Data acquisition and preprocessing parameters were matched with those of a previous investigation of the neural correlates of passive humor perception (Amir et al., 2015), to allow a comparison to humor generation. All fMRI images were scanned at USC’s Dana and David Dornsife Cognitive Neuroscience Imaging Center on a Siemens Trio 3T scanner with a standard 16-channel head coil. Each subject ran in a high-resolution T1-weighted structural scan using MPRAGE sequence. [Repetition time (TR) = 1100 ms, 192 sagittal slices, 256 × 256 matrix size, 1 mm × 1 mm × 1 mm voxels].

Functional images were acquired using an echo-planar imaging (EPI) pulse sequence with the parameters: TR = 2000 ms, TE = 30 ms, flip angle = 62°, 256 × 256 matrix size, in-plane resolution 3 × 3, 3 mm thick slices, 32 axial slices covering as much of the brain as possible, always including the Temporal Poles, but occasionally missing the superior rim of the primary motor and somatosensory cortices.

### Data Analysis

Preprocessing (3D motion correction using Trilinear interpolation, 3D spatial smoothing using a 4-mm full-width at half-max Gaussian filter, linear trend removal using a high-pass filter set to three cycles over the run’s length) was done with the Brain Voyager software package (Brain Innovation BV, Maastricht, The Netherlands). Statistical analysis was done using MATLAB scripts along with Brain Voyager, and Python. Motion corrected functional images were coregistered with the same session’s anatomical scan. Coregistered images were then transformed to Talairach coordinates and underwent statistical analysis.

Statistical analysis was based on a general linear model with a separate regressor for 12 TRs from the beginning of each trial type (so TR # 1 was recorded during the 2 s interval in which the instruction cue word was displayed). The six motion correction parameters (3D translation and 3D rotation) were included in the design matrix of the regression to eliminate any potential motion artifacts. We then conducted a whole-brain, random-effects group average analysis. We defined regions of interest (ROIs) using the data from all participants with different contrasts (HUM-MUN, HUM+MUN-2 × NOTH), TR-intervals (3–6, 7–10), as well as ROIs obtained in a previous experiment on passive humor appreciation. For the purpose of defining ROIs, we used different *p*-values for different (contrast, TR-interval) combinations, never higher than *p* = 0.001 uncorrected. *P* values were made more conservative in order to define smaller, well defined, ROIs as necessary (e.g., for the main contrast of HUM-MUN, we used *p* < 0.01 Bonferroni corrected). The ROIs were then used to compare activation in the different groups, and to assess whether the pattern activation in the region encoded the funniness of the caption.

### Evaluating Group Differences

The statistical measures of expertise effects (reported in the main text) were computed with a regression analysis of the average difference between HUM and MUN condition over the full duration of a trial and at peak activity (TRs 5–7), with age and sex included as regressors of no interest, with βs the (normalized) regression coefficients of expertise, *p* its significance, and *d* (Cohen’s *d*) the effect size of the difference between professional comedians and controls. For the regression analysis, we defined the variable “expertise” as: Controls = 0; Amateurs = 1; Professionals = 2. Note that we are treating the ordinal scale of expertise as interval for the purpose of the regression analysis, since it captures a surprisingly linear fit of activation in certain ROIs (**Figure 3**) – the expertise effects are further validated, however, by the *t*-test analysis of the activation differences between professionals and controls. For the sake of data exploration, we have further subdivided the Professionals groups to Professional Stand-Ups and Professional Improv Comedians, reasoning that since the task is closer to an improv than stand-up performance the latter group should show greater “expertise effects.” To illustrate that relationship we re-ran the regression analysis with Controls = 0; Amateurs = 1; Professional Stand-Ups = 2; Professional Improv = 3. That analysis resulted in similar βs and *p* values in the major ROIs, and the striking pseudo-linear relationship is illustrated in **Figure 3**.

### Assessing Funniness

Participants’ ratings of their own captions, as well as ratings obtained by the independent raters, were used to evaluate whether the ROIs obtained by contrasting the different conditions (e.g., HUM minus MUN) show a funniness magnitude effect, i.e., whether early activation in these regions (TRs 4–6) was related to how funny the subsequently generated caption would be. We chose early TRs in an attempt to target the process of generating a humorous caption rather than the evaluation of the fully generated caption. However, our previous work suggested that the two processes are intertwined – as the greater activation in temporal regions associated with successful linking of remote associations in joke generation/comprehension may also index the joke’s funniness (Biederman and Vessel, 2006; Amir et al., 2015). That said, it is unlikely that such activity only indexes passive humor appreciation as the peak of activity is early–before the humorous idea is fully formed (see Results)–and involves the high level semantic regions where remote associations are expected to converge meaningfully during humor creation (rather than mere classical reward regions activation). An ROI was considered to display a “funniness magnitude” effect if it was localized with the contrast HUM minus MUN, i.e., the ROI is humor selective, and greater activation in the ROI preceded the generation of funnier captions.

### Obtaining Independent Ratings

Following the fMRI scan, participants were presented with the images from their last 1–2 runs (time permitting) and were asked to recall and write down the captions that they had generated. 81 undergraduate students of the Department of Psychology were recruited to rate the recalled captions for course credit. Each spent an hour rating a fraction (typically a quarter) of the total number of captions on a 7-point scale for funniness, cleverness, and offensiveness. Ratings were normalized for each participant before all ratings were averaged.

### Eliminating “Double Dipping” Concerns

Whenever the same data are used for localizing ROIs and for statistical analysis within the ROI there is a concern about a potential bias due to non-independence (Kriegeskorte et al., 2009). In the present analysis, we first localized our ROIs using data from all 40 participants. For the main contrast of HUM minus MUN, a highly conservative threshold was used (*p* < 0.01, Bonferroni corrected) and the same regions were localized independently using only data from each group of participants (professionals, amateurs, and controls).

We then examined activation within those ROIs to observe between group differences in HUM minus MUN activation. While unlikely under the conservative threshold, this scheme could lead to non-independence concerns in the following manner: since the “controls” were the largest group of participants (*n* = 18) they might shift the boundary of the ROIs localized slightly to favor voxels in which activation is greatest in the control group. If that were the case, and there were no real group differences, a pattern in which the subsequent between group analysis showed greater activation in the control group might emerge from non-independence alone. We found the opposite effect of expertise in the temporo-occipital junction (TOJ; see results and **Figure 3**). To ensure these results were not an artifact of biased ROI boundaries we repeated the analysis using adjacent ROIs (in mPFC and STR) localized with independent data from a previously published study on passive humor appreciation (Amir et al., 2015), as no previous fMRI studies of humor creation exist. This analysis yielded nearly identical results in trend (PI < PS < A < C; see **Figure 3**), statistical significance and effect sizes – thus none of the group effects could be explained as an artifact of double dipping.

## Results and Discussion

### Behavioral Reaction Times

Reaction times (RTs) of the three groups (Professionals, Amateurs, and controls) as measured by the time for the key press for rating the funniness of the generated caption was 2.37 s greater for the HUM relative to the MUN condition; a difference that was significant for all levels of expertise (Supplementary Table S1). RTs for rating the captions were significantly longer for professional comedians relative to amateurs, apparently taking greater advantage of the 15 s trial duration, *t*(20) = 3.23, *p* < 0.005. No significant difference in RTs was observed between amateurs, and controls, *t*(24) < 1. The differences in RTs reported above were unlikely to have produced the fMRI differences between groups as the main ROIs were localized with the early peak of activation, which coincided for the two conditions (HUM and MUN). The RT gap between HUM and MUN did not differ significantly between the three groups (all *t*s < 1), and all group comparisons of BOLD activity were restricted to that obtained by first contrasting HUM vs. MUN activity within subjects. A related concern is that the difficulty of the HUM condition was greater than that of the MUN condition, as suggested by the longer RTs and that task difficulty rather than the requirement to be creative or humorous might explain any activation differences observed in the HUM vs. MUN contrast. However, as discussed below, several of the regions which showed greater activation in HUM than MUN also exhibited a “funniness magnitude” effect, that is, greater activation in those regions early in the trial correlated positively with funnier captions produced by the subject later in the trial (with funniness judged by independent raters) – rendering it more plausible that the regions were involved in the creative process.

### Neural Signature of Creating Humorous vs. Mundane Captions

Taken as a group the 40 participants showed significantly greater activation during HUM relative to MUN trials in bilateral striatum, mPFC, TOJ and primary visual cortex (V1; *p* < 0.01, Bonferroni corrected; see **Figure 2** and **Table 1**). A conjunction analysis of MUN and HUM conditions, contrasted with activation during the NOTH condition revealed additional activations in temporal regions – particularly the bilateral anterior temporal regions (*p* < 0.001, uncorrected; Supplementary Table S5). That activation occurred early in the trial, suggesting the regions’ involvement in the initial efforts to generate a humorous idea rather than the evaluation of the final product, or humor appreciation.

**Table 1:** ROIs as localized by the contrast of HUM minus MUN (Random Effects Analysis) with a threshold of *p* < 0.01 Bonferroni corrected (TR = 3–6).

ROI	NrOfVoxels	*X*	*Y*	*Z*	OWN	FUN	CLV	OFF
V1	4523	-3	-78	-11	P^∗∗^	P^∗^		
mPFC	637	-3	49	27				
STR	11985	-1	-3	6	P^∗∗^	P^∗^		
CER	539	0	-49	-32				
lTOJ	5978	-32	-78	-6	P^∗∗^	P^∗∗∗^	P^∗^	
rTOJ	3395	30	-82	5		P^∗∗∗^	P^∗∗^	

*With number of Voxels, Talairach coordinates and funniness magnitude effect for Professionals (P), Amateurs (A) and Controls (C). For self-rating (OWN), and independent ratings of funniness (FUN), cleverness (CLV) and offensiveness (OFF). Significance levels are: ^∗^*p* < 0.1, ^∗∗^*p* < 0.05, ^∗∗∗^*p* < 0.01. Key: r, right; l, left; V1, primary visual cortex; mPFC, medial prefrontal cortex; CER, cerebellum; STR, striatum; TOJ, temporo-occipital junction.*

Most studies of passive humor appreciation have reported involvement of high-level semantic regions in the temporal lobes (Vrticka et al., 2013) and we have previously suggested that is the region where remote associations converge meaningfully when “getting” a joke (Amir et al., 2015). The present results suggest that those regions are involved in creating humor as well, albeit with a different time course (see section Active Humor Creation vs. Passive Humor Appreciation). The mPFC has been implicated in most studies of creativity (Liu et al., 2015), as well as in some studies of humor appreciation (e.g., Amir et al., 2015). It appears to be involved in humor creation as well, but is likely not the source of humorous ideas (see General Discussion). The greater activation of V1 during the HUM condition may reflect a greater engagement of visual search for aspects of the drawing, perhaps an incongruity, with a comedic potential. In the case of passive humor appreciation (Watson et al., 2007), greater visual cortex activation was reported for visual gags (relative to non-humorous visual stimuli), but not to language gags – this additional visual activation was suggested by Watson et al. (2007) to reflect the resolution of the punchline, but such reactivation of visual areas occurs for any reinterpretation of a visual stimulus, humorous or not (Sterzer et al., 2009; Amir et al., 2015).

### Independent Funniness Ratings Associated with Greater Early Activation in Temporal Regions and Striatum

Amir et al. (2015) found that temporal regions, TOJ and TP, exhibited a funniness “dose response.” That is, activity in those regions were greater for humorous than non-humorous stimuli and for the humorous stimuli, the same regions responded more strongly to the instances with higher funniness ratings. Similarly, in professional comedians we observed that early in the trial, the generation of funnier captions elicited greater activity in the striatum, bilateral TOJ, and other temporal regions (but not in mPFC). The regions were localized by subtracting MUN from HUM trials, that the activation was even greater for the funnier captions in those regions can be described as a funniness magnitude effect. The relationship held whether funniness was evaluated based on the comedians’ own ratings or by independent raters (only rated captions from the HUM condition were included in this analysis; see **Table 1**, Supplementary Table S5). The funniness magnitude effect was observed early in the time-course of the trial (TRs 4–6), suggesting it reflects the process of creating the humorous caption rather than the evaluation of its final product. Controls and amateurs, however, showed no funniness magnitude effect in the regions localized by the contrast HUM minus MUN. Controls did show such correlations in some of the regions localized by the conjunction of HUM and MUN (Supplementary Table S5). The funniness magnitude effect cannot be explained away by mere increased effort or engagement as that would entail an increase of activity in all regions of the network (defined by the contrast HUM minus MUN, which includes mPFC) during the generation of the funnier captions, which was not the case as mPFC showed higher activation, on average, during the generation of the *less* funny captions.

Of the few MRI creativity studies, only a handful attempted to correlate the quality of the creative product with BOLD activation. The results of those studies are difficult to compare as they used different measures for “quality” (from novelty and complexity to “craft”) with some of those studies (ours included) correlating the trial-by-trial quality scores to activity, while others approximated it with subjects’ general creativity scores on a separate creativity task. Nevertheless, a pattern emerges suggesting that the quality of different creative tasks correlate with activity in different regions: complexity of pianists’ improvisation correlates with activity in pre-SMA cortex (Bengtsson et al., 2007), creativity of rhythm improvisation is associated with bilateral prefrontal cortex and right insula (Villarreal et al., 2013), creative writing with the left fronto-temporal network (Shah et al., 2013), free style rap performance with activity in medial temporal regions, posterior cingulate cortex and left mPFC (Liu et al., 2012), and skillful drawing with changes in prefrontal white matter connectivity and a distinct pattern of activation in temporal, motor and prefrontal cortices (Schlegel et al., 2015). Even within the same imaging study during a poetry creation task, different measures of quality, craft and the linguistic creativity evident in the poetry, were associated with different networks involving mPFC and dlPFC, respectively (Liu et al., 2015). The lack of consistency suggests that there is no one region acting as a general creativity fount so that greater activity in that region correlates with creative products that are of higher quality. Distinct regions appear to play this role for distinct creative tasks. Here we find the quality of humor creativity, i.e., funniness, is associated with temporal and striatal activity.

### Neural Correlates of Comedic Expertise

We observed a clear function of comedic experience/talent throughout the trial so that HUM minus MUN activity in the mPFC (regression coef. β = -0.55, *p* < 0.01, Cohen’s *d* = 1.43) and striatum (right: β = -0.43, *p* < 0.05, *d* = 1.07; left: β = -0.46, *p* < 0.05, *d* = 1.47) was greatest for Controls than Professional comedians with Amateurs falling in between. The reverse was true in the right TOJ with peak activation (β = 0.31, *p* < 0.05, *d* = 1.05) greatest for Professionals and smallest for Controls. The left TOJ exhibiting a similar pattern that failed to reach significance. Of the Professionals, Improv Comedians showed even greater activation in TOJ on average and lower activation in mPFC and STR than the Stand-Up Comedians. That result is in line with the trend set by Controls vs. Amateurs and the Professionals group as a whole, since the experimental task is more similar to an improv comedy scene than stand-up, so Improv Comedians would be judged to possess the greatest level of expertise on the experimental task (**Figure 3**). These results cannot be explained by non-independent selective analysis biases (a.k.a. “double dipping”; see Materials and Methods). We propose that the temporal regions are where remote associations converge meaningfully in the process of constructing the joke, while mPFC directs the process of deliberate search in a top-down manner, and that the expertise/talent effects suggest that with greater comedic expertise less involvement of control processes (localized in mPFC) is needed as the Comedians reap the fruits of their spontaneous associations (see General Discussion).

The Comedians had a smaller proportion of females than the controls, a statistic that roughly reflects the high proportion of males among comedians (Greengross et al., 2012). In computing the beta values above, age and sex were controlled for as regressors of no interest, and typically showed no significant correlations with the ROIs’ activations, with the one exception of sex in rTOJ. During humorous caption generation, maleness was positively correlated with rTOJ activity, in a similar fashion to comedic expertise (β = 0.35, *p* < 0.05).

### Active Humor Creation Vs Passive Humor Appreciation

**FIGURE 4:**
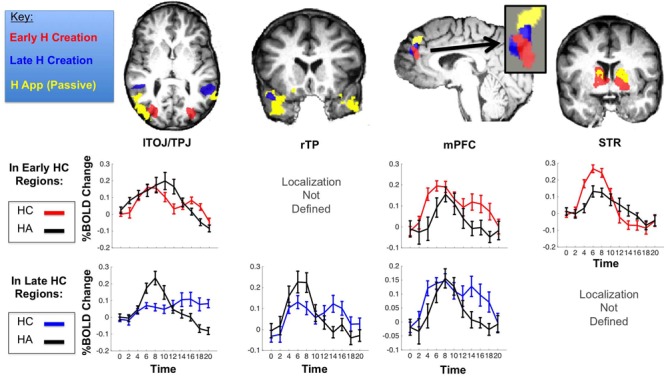
**Top: ROIs selective to humor creation (HC) early in the trial (red), late in the trial (blue), and in passive humor appreciation (HA) – overlaid from a previous study (Amir et al., 2015; yellow).** Graphs show activation of HUM minus MUN in early (**Top**, in red) and late (**Bottom**, blue) HC areas, with passive HA activation (data obtained from Amir et al., 2015) in the same regions in black.

**FIGURE 5:**
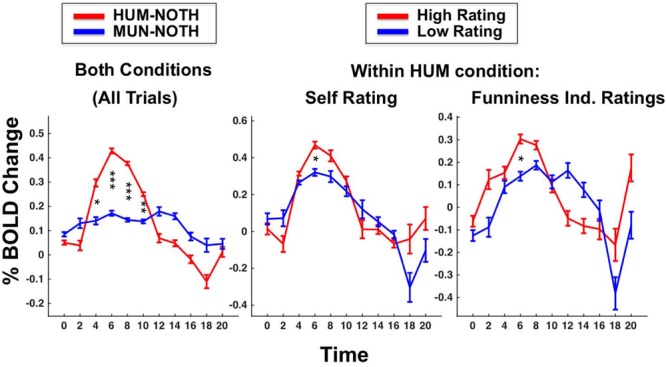
**Striatum activation in professional comedians is greater in HUM then MUN condition **(Left)**, and within the HUM condition is greater prior to the generation of funnier captions, as rated by the participant **(Middle)** or independent raters **(Right)**.** Stars indicating significance: ^∗^*p* < 0.05, ^∗∗^*p* < 0.01, ^∗∗∗^*p* < 0.001.

While passive humor appreciation has been extensively studied (Vrticka et al., 2013; Amir et al., 2015), the present study is the first yet to explore humor creation. The regions we found to be involved in humor creation (or, at least, adjacent regions) have been implicated in passive humor appreciation in some of the previous studies (including our own, Amir et al., 2015). However, there are distinct time course differences for passive vs. active humor processing. In this section, we characterize the differences between humor creation and appreciation by comparing the current results to those of Amir et al. (2015), as well as comparing activity in the early vs. late stages of humor creation. Humor creation, at least with improv comedy, has another favorable feature facilitating its MRI investigation: it is a well-defined cognitive event, long enough to tease apart both in its early (**Figures 2**–**5**) as well as its later stages (**Figure 4**). A whole-brain contrast of HUM minus MUN late in the trial (Supplementary Table S6; **Figure 4**) revealed that activation shifted from bilateral TOJ toward the TPJ and more anterior temporal regions, closer to regions that have previously been identified as selective for passive humor appreciation (Vrticka et al., 2013; Amir et al., 2015). During humor appreciation, the time-course of temporal activation peaks early and declines rapidly relative to humor creation, presumably reflecting that “getting a joke” generally would occur more quickly—and end earlier—than the act of creating a joke. In contrast to the shorter activity peak of passive humor appreciation, active humor creation resulted in a gradual increase in anterior temporal and TPJ activation throughout the trial, suggesting the gradual construction of comedic meaning via the discovery and linkage of remote associations.

### Funniness Ratings

Eighty-one independent raters (that did not take part in the fMRI experiment) rated the captions obtained in the fMRI experiment (each rated about a quarter of the captions with the ratings normalized within by the individual rater prior to their compilation). Reassuringly, all subjects self-rated their captions as funnier in the HUM that in the MUN trials (Supplementary Table S2) and the independent raters also rated HUM captions significantly higher for all groups of participants (Supplementary Table S3), with overall significant covariance between subjects’ own ratings and independent raters ratings (Supplementary Table S4). The differences in self-ratings between HUM and MUN trials were similar among the three groups (all *t*s < 1), but independent raters saw a greater difference in funniness between HUM and MUN trials for the controls compared to the professional comedians [*t*(24) = 2.10, *p* < 0.05, Cohen’s *d* = 0.95]. Taken together, it appears that regardless of expertise, when generating captions, people (at least intelligent adult Americans) have some degree of control over the funniness of the outcome, but professional comedians may be less successful in suppressing funny ideas when asked to generate mundane captions (as also volunteered by several professional comedians’ introspections during the debriefing).

In contrast to the expectation that at least professional comedians should produce funnier captions than the other groups, the funniness scores from the independent raters did not distinguish among the three participant groups (professionals, amateurs or controls), all *t*s < 1. This should not in any way cast doubt as to the “expert” classification of our professional comedians, as having a Netflix Comedy Special or gaining membership in the Groundlings are extraordinarily competitive achievements. Rather, the nature of our drawings–cartoons in which any incongruous or funny elements were removed–resulted in a particularly difficult setting for the comedians who seek an incongruous or unusual element in a scene as the point of departure for their humor (Mankoff, 2002). That said, the incongruity and humor-free nature of our stimuli has the advantage of not confounding the neural correlates of the participants generating humorous ideas from those of appreciating the humor in the prompt itself. The effects of incongruity in the settings would appear to be a problem worth studying for understanding the nature of humor creativity. The average (HUM/MUN) number of captions remembered differed among professionals (7.2/6.8), amateurs (12.1/11.9) Controls (9.5/10.6). Not much is to be made of those group differences as they partially resulted from extraneous factors such as professionals having a busier schedule and so less time to spend after the experiment remembering captions. To ensure the group comparisons of funniness were not biased by the number of captions remembered (e.g., if participants who remembered fewer captions remembered the funnier ones) the analysis was repeated with the funniest 5, 3, and 1 captions with similar results.

## General Discussion

Professional comedians, amateurs, and controls generated captions to NewYorker cartoons, revealing a network of regions–including bilateral TOJ, mPFC and the striatum–that exhibited greater activation during humorous (HUM) vs. mundane (MUN) caption generation.

Many of the MRI studies of humor appreciation have found greater activation in some high-level semantic regions in the temporal lobes (e.g., TOJ, TPJ, TP, STS) during the humorous condition (Vrticka et al., 2013). These regions, where information converges from diverse lower level regions (Man et al., 2013) are likely the site were remote associations converge meaningfully during the comprehension of a humorous product. There is greater activation in those regions for humorous than non-humorous discoveries/insights, as well as a dose response function, i.e., among humorous stimuli, those rated funnier by the participants produce greater activation in those regions (Amir et al., 2015). Based on the discovery of a gradient of μ-opioid receptors (Lewis et al., 1981) peaking in associative regions in the temporal lobes, activation in semantic temporal association regions (TMP) has been found to be pleasurable and likely a factor in the feeling of mirth itself (Biederman and Vessel, 2006). That temporal associations tend to elicit positive emotions has been documented in preferences for scenes (Yue et al., 2007), simple shapes (Amir et al., 2011) and jokes (Amir et al., 2015; Amir, 2016). Thus, it was not surprising that we found greater activation there during humor creation during which, presumably, remote associations are generated and linked in the process of joke construction. While temporal activity is observed during both humor appreciation and creation, peak activity during creation is observed in adjacent but more posterior regions and shows a temporal pattern of continuous gradually increasing activity throughout the trial in contrast to the shorter rise and fall of activation during humor appreciation, presumably corresponding to the act of “getting the joke.” Analogous to the temporal lobes’ “dose response” of passive humor appreciation, our professional comedian participants show a “funniness magnitude” effect so that greater activation in the temporal lobes early in the trial correlates with the generation of funnier captions later in the trial. Finally, the contrast HUM-MUN yielded greater activity in temporal regions for professional comedians followed by amateurs and controls in declining order, suggesting reliance on those regions during comedy creation increases with experience (and/or talent). Taken together the findings suggest that the temporal regions are likely where comedic meaning is represented and constructed.

Since all drawings depicted people interacting, could the temporal activity during the HUM condition reflect a greater engagement of theory of mind (Saxe and Kanwisher, 2003) rather than convergence of remote associations? Since both the HUM and MUN used the same drawings and required participants to generate a statement one of the characters would say in the situation, contrasting the two conditions is expected to control for theory-of-mind effects on activation, unless during humor creation participants engage in theory of mind processing to a greater extent. While the current paradigm does not allow us to determine the exact cognitive processes indexed by the temporal activity (be it convergence of remote associations, theory of mind, or other processes), it appears to be related to humor creation as it is greater for humorous than mundane caption generation, and correlates positively with caption’s funniness as well as participants’ comedic expertise.

The mPFC is the region most consistently reported in fMRI creativity studies (Dietrich and Kanso, 2010; Liu et al., 2015; Saggar et al., 2015), in jazz improvisation (Limb and Braun, 2008), rap improvisation (Liu et al., 2012), and story generation (Howard-Jones et al., 2005), but also in problem solving tasks that would appear to require less creativity, such as anagram solutions (Aziz-Zadeh et al., 2009). The mPFC’s role is likely to extend cognitive control over the creative process (Ridderinkhof et al., 2004; Passingham et al., 2010). However, our findings suggest the mPFC might not be the *source* of creative ideas as it does not show a funniness magnitude effect (i.e., there was no correlation between activity there and caption funniness, in contrast to the temporal regions) and is less activated in professional comedians relative to amateurs and controls. The mPFC showed *less* activation, while the TOJ (as well as some anterior temporal regions), were *more* active in professional comedians, relative to controls, suggesting professionals rely more on the spontaneous flow and linking of associations in the temporal regions, with less mPFC engagement for deliberate search. This result is in line with Schlegel et al. (2015) who found that fractional anisotropy in prefrontal white matter progressively decreased with visual art training, suggesting a reorganization of connectivity to the region. The pattern of decreased mPFC and increased temporal activity may be the translation to neuroscience language of the most common advice offered by improv comedy coaches: “get out of your head.”

The striatum is part of the classical reward system and is activated in response to any pleasurable stimulus, including humor as well as other forms of art (Vessel et al., 2012). Unlike the case of humor appreciation (Amir et al., 2015), where striatal activation follows or coincides with activation of temporal regions, peak striatal activation preceded the peak of temporal activation in the case of humor creation. The striatum also showed a correlation between early activation and the creation of funnier captions in professional comedians (**Figure 5**). Whether the magnitude of the funniness effect in the striatum of professional comedians reflects an on average accurate expectation that the caption they will generate later in the trial will be funnier or is playing a more causal role, e.g., by helping the retrieval of associations with a greater potential for humor (Scimeca and Badre, 2012) remains to be determined. The latter interpretation is consistent with a common comedy coaches’ advice: “have fun and you will be funnier.” Some of the alternative explanations for the expertise effect on striatal activation, e.g., that comedians have a more depressive emotional style or that comedians are adapted to the reward of humor creation, are inconsistent with the finding that the BOLD response of professional comedians to the HUM condition by itself (i.e., not contrasted with MUN) is as high as that of controls.

## Conclusion

Humor creation is marked by activation in a network of regions including mPFC, the striatum, and temporal regions. Only activation in the temporal regions exhibited both a positive correlation with expertise as well as a “funniness magnitude” effect (greater activation early in the trial predicts a funnier caption at trial’s end) suggesting the temporal regions are a likely source of the humorous ideas. While greater mPFC activity was observed during humor creation (relative to generation of mundane captions) the activity decreased with occupational experience, suggesting that while mPFC might help to direct the search through association space taking place in the temporal regions, such intervention is needed less for more experienced comedians who, to a greater extent, reap the fruits of their spontaneous associations.

## Author Contributions

OA and IB conceived and designed the experiment, and written the manuscript. OA conducted the experiment and data analysis.

## Conflict of Interest Statement

The authors declare that the research was conducted in the absence of any commercial or financial relationships that could be construed as a potential conflict of interest.
