# Supplementary material for: The Neural Correlates of Humor Creativity
Source: Front Hum Neurosci. 2016 Nov 25;10:597. doi: 10.3389/fnhum.2016.00597 (PMC5122582; doi:10.3389/fnhum.2016.00597)
Supplement: Supplementary file 1 [file Table_1.docx]

**Sup. Table 1.** RTs of the two conditions for the 3 levels of expertise/talent.

|  | **Humor** | **Mundane** | **DF** | ***t*** | ***P*<** |
| --- | --- | --- | --- | --- | --- |
| Professionals | 12.66 | 10.53 | 12.00 | 3.94 | .005 |
| Amateurs | 10.17 | 8.05 | 8.00 | 2.15 | .07 |
| Controls | 9.65 | 6.81 | 16.00 | 5.64 | .001 |

**Sup. Table 2.** *Self-Ratings* of the two conditions for the 3 levels of expertise/talent.

|  | **Humor** | **Mundane** | **DF** | ***t*** | ***P*<** |
| --- | --- | --- | --- | --- | --- |
| Professionals | 2.50 | 1.67 | 12.00 | 3.17 | .01 |
| Amateurs | 2.76 | 1.70 | 8.00 | 2.67 | .03 |
| Controls | 2.52 | 1.84 | 16.00 | 2.04 | .06 |

**Sup. Table 3.** Funniness ratings of *independent raters* of the two conditions for the 3 levels of expertise/talent.

| Group | HUM M (SD) | MUN M (SD) | T | DF | P< | Cohen's d |
| --- | --- | --- | --- | --- | --- | --- |
| Amateurs | 4.40 (.60) | 3.88 (.53) | 2.01 | 7 | 0.04 | 1.00 |
| Professionals | 4.50 (.34) | 3.96 (.49) | 2.90 | 9 | 0.01 | 1.60 |
| Controls | 4.51 (.38) | 3.56 (.15) | 8.59 | 15 | 0.001 | 6.44 |

**Sup. Table 4.** Average r^2 values for correlations between self-rating of captions (OWN) and independent ratings of those captions for funniness (FUN) cleverness (CLV) and offensiveness (OFF). All the r^2 values in the table are significantly greater than 0 (** *p* < .01; * *p* < .05).

|  | FUNxOWN | CLVxOWN | OFFxOWN |
| --- | --- | --- | --- |
| Professionals | .22** | .23** | .20* |
| Amateurs | .35** | .38** | .25** |
| Controls | .10** | .10** | .12** |

**Sup. Table 5.** ROIs as localized by the conjunction of HUM and MUN minus twice NOTH with a threshold of p < .001 uncorrected (TR=3-6). With number of Voxels, Talairach coordinates and funniness magnitude effect for Professionals (P), Amateurs (A) and Controls (C). For self-rating (OWN), and independent ratings of funniness (FUN), cleverness (CLV) and offensiveness (OFF). Significance levels are: * *p* < .1, ** *p* < .05, *** *p* < .01. Key: r – right; l – left; m – medial; Neg – negative (i.e. the region was localized by a significantly greater activity for the NOTH condition); TOJ – temporo-occipital junction; STR – striatum; Sup – superior; Ant – anterior; TP – temporal pole; AMG – amygdala; TPJ – temporal parietal junction; CER – cerebellum; ACCdSPM – anterior cingulate cortex/dorsal supplementary motor cortex; PCC – posterior cingulate cortex; vmPFC – ventromedial prefrontal cortex.

| **ROI** | **NrOfVoxels** | **X** | **Y** | **Z** | **OWN** | **FUN** | **CLV** | **OFF** |
| --- | --- | --- | --- | --- | --- | --- | --- | --- |
| Neg rParietal supramarginal gyrus | 10571 | 51 | -43 | 31 |  | P** |  | P* |
| Neg rAntTPJ | 1758 | 47 | -10 | 3 |  | P** |  |  |
| Neg lAntFrontal | 620 | 38 | 44 | 11 |  | P** |  | P* |
| Neg rDLPFC | 649 | 32 | 20 | 38 |  | P* |  |  |
| Neg PCC | 2940 | 4 | -34 | 36 | P* |  |  |  |
| ACCdSPM | 26325 | -5 | 21 | 42 | P* |  | C* |  |
| mCER | 2337 | -2 | -48 | -29 |  |  |  |  |
| vmPFC | 1773 | -2 | 48 | -6 |  | P** |  | P* |
| Cuneus | 3358 | -7 | -55 | 13 |  |  | C** | C** |
| lAMG | 380 | -40 | -14 | -23 |  |  |  |  |
| Neg_lParietal_ supramarginal_gyrus | 948 | -60 | -42 | 33 |  | C*** | C*** | C** |
| lSupAntTemporal | 4911 | -52 | -8 | -13 |  |  | C* |  |
| lTP | 8737 | -49 | 11 | -17 |  | P** |  |  |
| lFrontal | 21542 | -45 | 21 | 6 | P* |  |  |  |
| lSupFrontal | 16089 | -42 | 0 | 34 |  | C** | C** |  |
| lSTR | 14276 | -17 | -7 | 5 | P** |  | C* |  |
| rSTR | 8273 | 13 | -4 | 7 |  | P** | C* | P* |
| rSupAntTepmporal | 6234 | 44 | -10 | -16 | P* |  |  |  |
| rPostSupParietal | 9542 | 43 | -42 | -1 | P** |  |  |  |
| rTOJ | 32003 | 29 | -81 | -14 | P** | P** |  |  |
| lTOJ | 17677 | -38 | -75 | -11 | P* | P** | P* | P* |

**Sup. Table 6.** Late humor generation ROIs (TR=7-10) as localized by the contrast of HUM minus MUN (Random Effects Analysis) with a threshold of p < .001 uncorrected. With number of Voxels, Talairach coordinates and funniness magnitude effect for Professionals (P), Amateurs (A) and Controls (C). For self-rating (OWN), and independent ratings of funniness (FUN), cleverness (CLV) and offensiveness (OFF). Significance levels are: * *p* < .1, ** *p* < .05, *** *p* < .01. Key: r – right; l – left; m – medial; Neg – negative (i.e. the region was localized by a significantly greater activity for the MUN relative to HUM condition, late in the time course of the trial); TOJ – temporo-occipital junction; TPJ – temporal parietal junction; STR – striatum; Sup – superior; Inf – inferior; TP – temporal pole; CER – cerebellum; ACCdSPM – anterior cingulate cortex/dorsal supplementary motor cortex; vmPFC – ventromedial prefrontal cortex; dlPFC – dorsolateral prefrontal cortex; LO – lateral occipital.

| **ROI** | **NrOfVoxels** | **X** | **Y** | **Z** | **OWN** | **FUN** | **CLV** | **OFF** |
| --- | --- | --- | --- | --- | --- | --- | --- | --- |
| lInfFrontal | 23962 | -42 | 15 | 8 | P** |  |  |  |
| rCER | 14212 | 27 | -54 | -26 |  |  |  |  |
| ACCdSMC | 13641 | -3 | 5 | 46 | P** |  | C* |  |
| rLO | 12104 | 31 | -85 | -9 | P** | P** |  |  |
| lMotor/  SupFrontal | 11223 | -42 | -4 | 39 |  | C** | C** | C* |
| lLO | 10895 | -33 | -82 | -13 | P* | P** | P* |  |
| lTOJ/TPJ | 3123 | -53 | -48 | 6 | P** | P* |  |  |
| lMotor | 2909 | -38 | -26 | 48 |  | P*** C* | P* C** | P** C** |
| mPFC | 2595 | -6 | 49 | 32 |  |  |  |  |
| rFrontal | 2385 | 34 | 15 | 1 | P* |  |  |  |
| rTOJ/TPJ | 1671 | 50 | -39 | 4 | P** |  |  |  |
| NeglSupFrontal | 1433 | -27 | 19 | 37 | P* |  |  |  |
| ldlPFC | 968 | -27 | 41 | 27 |  |  |  |  |
| BrainStem | 491 | -3 | -21 | -15 | P* |  |  |  |
| rMotor | 482 | 49 | -6 | 42 |  |  | C** |  |
| rTP | 468 | 44 | 16 | -17 | P** |  |  |  |
| rSTR | 351 | 10 | -10 | 4 | P** A* | C* | C* |  |
| rdlPFC | 329 | 55 | 17 | 19 | P* | P** | P** | P* |
| Neg_Precuneus | 51072 | 2 | -40 | 40 |  | P** | C* |  |
| Neg_Cuneus | 45830 | 0 | -74 | 14 |  | P** | C* | P* |
| Neg_vmPFC | 30859 | 1 | 18 | -3 |  | P** | P** | P* |
| Neg_rInsula | 24685 | 44 | -19 | 12 |  |  |  |  |
| Neg_lInsula | 5351 | -45 | -22 | 7 | P** |  | C* |  |
| NeglTP | 765 | -57 | -2 | -29 |  | A** | A** |  |
